# A new millipede of the family Ammodesmidae found in central Africa (Diplopoda, Polydesmida)

**DOI:** 10.3897/zookeys.483.9150

**Published:** 2015-02-19

**Authors:** Didier VandenSpiegel, Sergei I. Golovatch

**Affiliations:** 1 Royal Museum for Central Africa, B-3480 Tervuren, Belgium; 2 Institute for Problems of Ecology and Evolution, Russian Academy of Sciences, Leninsky prospekt 33, Moscow 119071 Russia

**Keywords:** *Ammodesmus*, taxonomy, new species, key, sex dimorphism, Democratic Republic of the Congo

## Abstract

The first species of the small Afrotropical family Ammodesmidae discovered in central Africa (Democratic Republic of the Congo) belongs to the genus *Ammodesmus* Cook, 1896, which was hitherto known only from two species in western Africa. A key is given to incorporate *Ammodesmus congoensis* sp. n., a species also showing an evident sex dimorphism: ♂ densely hirsute, ♀ with much longer and sparser tergal setae.

## Introduction

The Afrotropical millipede family Ammodesmidae has hitherto been known by only two genera: *Ammodesmus* Cook, 1896, with two species from western Africa (Guinea, Liberia and Ivory Coast), and *Elassystremma* Hoffman & Howell, 1981, with four species from eastern Africa (Kenya, Tanzania and Malawi) (VandenSpiegel and Golovatch 2012). The huge geographical gap between these genera has long remained enigmatic as to which ammodesmids could possibly occur there (e.g. Hoffman and Howell 1981, Hoffman 1993).

At the moment, *Ammodesmus* contains *Ammodesmus granum* Cook, 1896, the type species, from several places in Guinea, Liberia and Ivory Coast, including Mt. Nimba, as well as *Ammodesmus nimba* VandenSpiegel & Golovatch, 2012, from a single forest patch on Mt. Nimba, at the border between these three countries. This genus is easily distinguished by the particularly small size of its species (adults < 3.0 mm in length) and, especially, the strongly modified last legs in both sexes, in which the tibia is supplied with a long flagellum distodorsally (VandenSpiegel and Golovatch 2012).

Examination of some old material from the Democratic Republic of the Congo (formerly, first Belgian Congo, then Zaire) revealed the first ammodesmid samples. These represent a new species of *Ammodesmus* which is described here. This discovery shows a far vaster distribution of *Ammodesmus* in western and central Africa than previously known. In addition, a key is provided to all three known species of the genus.

## Material and methods

The material examined belongs to the collection of the Royal Museum for Central Africa (MRAC), Tervuren, Belgium, with only a few duplicates shared with the collection of the Zoological Museum, State University of Moscow (ZMUM), Russia, as indicated hereafter. All samples are stored in 70% ethanol. Specimens for scanning electron microscopy (SEM) were air-dried, mounted on aluminium stubs, coated with gold and studied using a JEOL JSM-6480LV scanning electron microscope.

## Description

### Ammodesmus congoensis sp. n.

Taxon classificationAnimaliaPolydesmidaAmmodesmidae

http://zoobank.org/FD3B5F5E-37B0-433B-BDB9-32DF3A4426AC

Figs 1 , 2 , 3

#### Type material.

Holotype ♂ (MRAC 20150), Democratic Republic of the Congo, Parque National Albert, secteur Sud, Berlese extraction, 1956, leg. R.P. Celis. Paratypes: 3 ♂ (MRAC 20150), same data, together with holotype; 5 ♀ (MRAC 20151), same data; 1 ♂, 1 ♀ (ZMUM p2441), same data; 2 ♀ (MRAC 20171), same data; 4 ♂, 2 ♀ (MRAC 20149), same data; 1 ♂, 1 subadult ♀ (MRAC 20274), same data; 1 ♂ (MRAC 20201), same data; 5 ♂, 16 subadult & earlier instar ♂, 1 ♀ (MRAC 20294), same data.

#### Name.

To emphasize the provenance of the new species from Congo.

#### Diagnosis.

Minute polydesmidans (length 1.5–2.1 mm, width 0.6–0.8 mm) with 18 or 19 body segments in both sexes, missing ozopores, simple biramous gonopods, and evident sexual dimorphism in tergal structure: metaterga in ♂ very densely pilose all over, with only few longer setae, devoid of a median transverse gutter, whereas ♀ metaterga with a deep transverse gutter in anterior third supporting a single row of ca 10–18 long setae positioned at gutter’s bottom.

#### Description.

**Figure 1.**
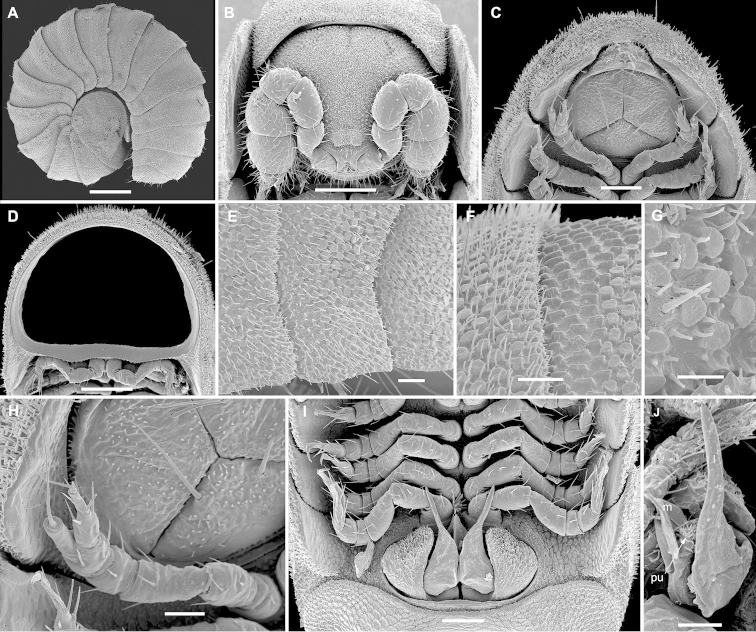
*Ammodesmus congoensis* sp. n., a 18-segmented ♂ paratype. **A** habitus of male, lateral view **B** head, ventral view **C** posterior part of body, caudal view **D** caudal view of a midbody segment, caudal view **E** midbody paraterga, lateral view **F**, **G** tegument texture, dorsal view **H** last left leg, lateral view; I both gonopods *in situ*, ventral view **J** left gonopod, submesal view. Scale bars: **A** = 200 µm; **B, D** = 100 µm; **C, I** = 50 µm; **E, F** = 20 µm; **G, J** = 10 µm. (**l** lateral finger-shaped branch, **m** mesal finger-shaped branch, **pu** pulvillus).

Length of adults 1. 5–2.1 mm, width 0.6–0.9 mm, ♂ usually a little smaller than ♀. Adult body with 18 or 19 segments (17+1+T) (♂, ♀). Entire dorsal surface covered with a thin layer of secretions (= cerategument) borne by microvilli, often also a dirt crust, under which the body integument is uniformly yellowish. Body shape as in Figs 1A, 3A, with caudal body end tapering towards a relatively small telson, the latter not being concealed by last paraterga (Figs 1C, 3F). Head small, only partly concealed under front edge of collum (Figs 1B, 3B); upper half of head densely granulate, lower part smooth and densely setose. Interantennal isthmus about as wide as antennomere 1 or antennal socket diameter (Fig. 1B). Antennae very strongly clavate due to subequal antennomeres 5 and 6 (Fig. 1B). Collum subquadrate, slightly impressed along longitudinal axis (Fig. 3B); tergum 2 as usual, hypertrophied, with strongly enlarged, spatuliform paraterga concealing the head in lateral view (Figs 1A, 3A, C). Limbus nearly smooth, at most with sparse, well separated, short spicules. Metaterga either regularly convex and uniformly very densely pilose throughout, with only few scattered longer setae (♂, including juveniles) (Fig. 1E, F, G) or nearly smooth, showing a deep transverse gutter in anterior 1/3, the latter supplied with ca 10–18 especially long, microspiculate setae at bottom and bearing a small pit marking ventral end of gutter about midheight of paraterga (♀, including juveniles) (Fig. 3A, D). Prozonae and stricture thereafter beset with round scales with peripheral hair-like cuticular outgrowths (Fig. 1F, G). Paraterga vertical, continuing the highly convex outline of dorsum, their ends rounded, projecting far below venter/coxae (Figs 1E, 3E), increasingly angular towards telson (Fig. 3E). Anteroventral edges of paraterga 3 to 15 each with a notch forming a groove for paratergum 2 to hinge into during volvation. Ozopores absent. Telson small (Figs 1C, 3F). Hypoproct triangular (Figs 1C, 3F).

Legs rather slender, but short, barely reaching tips of paraterga (Fig. 1C, I); femora and tarsi subequal in length, longer than other podomeres; claw normal, simple, very slightly curved ventrad; last pair of ♂ and ♀ legs modified, typical of *Ammodesmus*, with a long distodorsal flagellum borne on a stump on tibia (Figs 1H, 3G).

**Figure 2.**
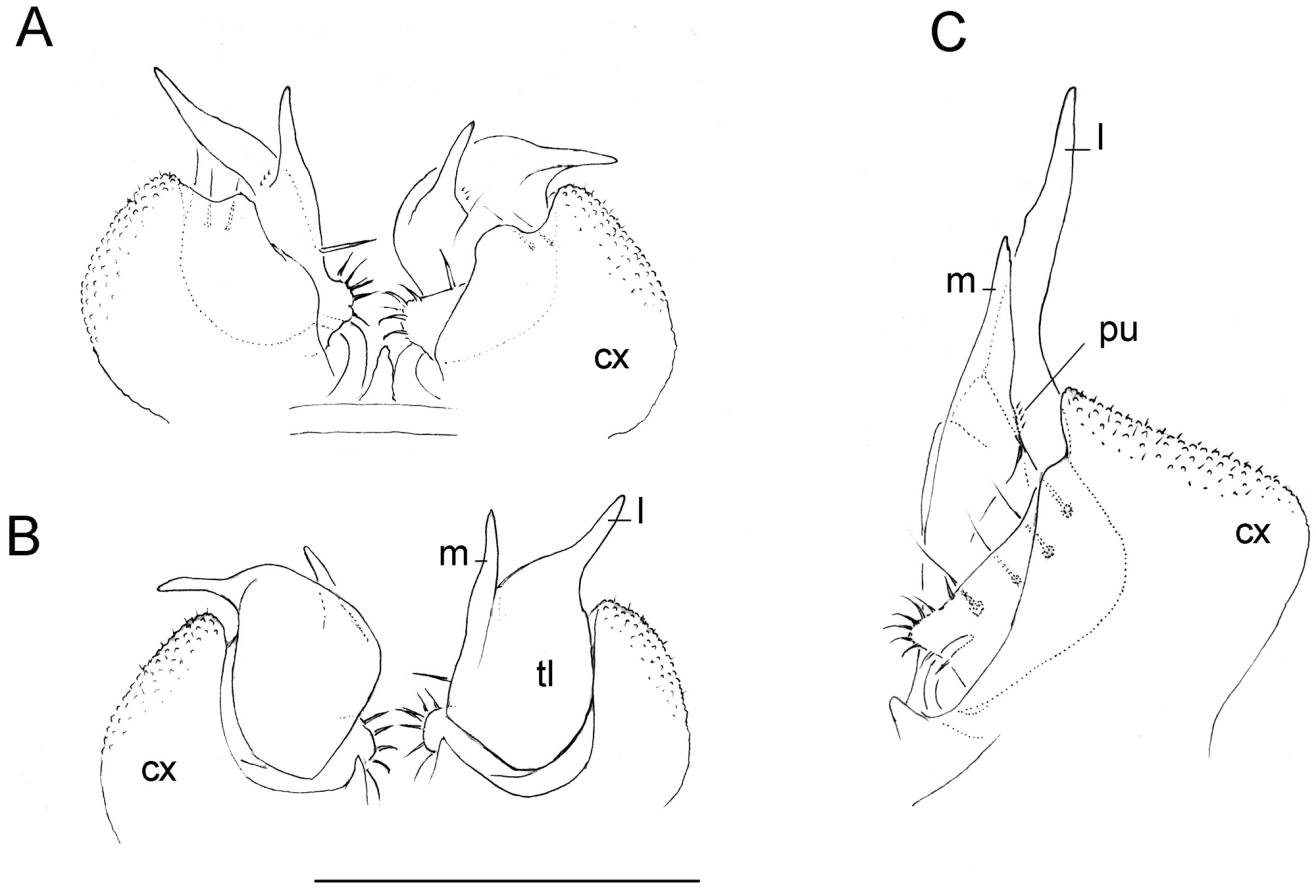
Gonopods of a 18- (**A, B**) and a 19-segmented ♂ paratype (**C**). **A, B** both gonopods *in situ*, frontal and caudal views, respectively **C** left gonopod, frontal view. Scale bar: 1.0 mm (**cx** coxae, **l** lateral finger-shaped branch, **m** mesal finger-shaped branch, pu pulvillus, **tl** telopodite).

Gonopods (Figs 1I, J, 2) relatively simple. Coxae small, globulose, micropapillate and microsetose laterally. Telopodite long and well-exposed beyond gonocoxae, divided into two finger-shaped branches: a shorter mesal (**m**) and a longer lateral (**l**); a small hairy pulvillus (pu) marking the orifice of a short seminal groove at base between **m** and **l.** A solenomere absent.

**Figure 3.**
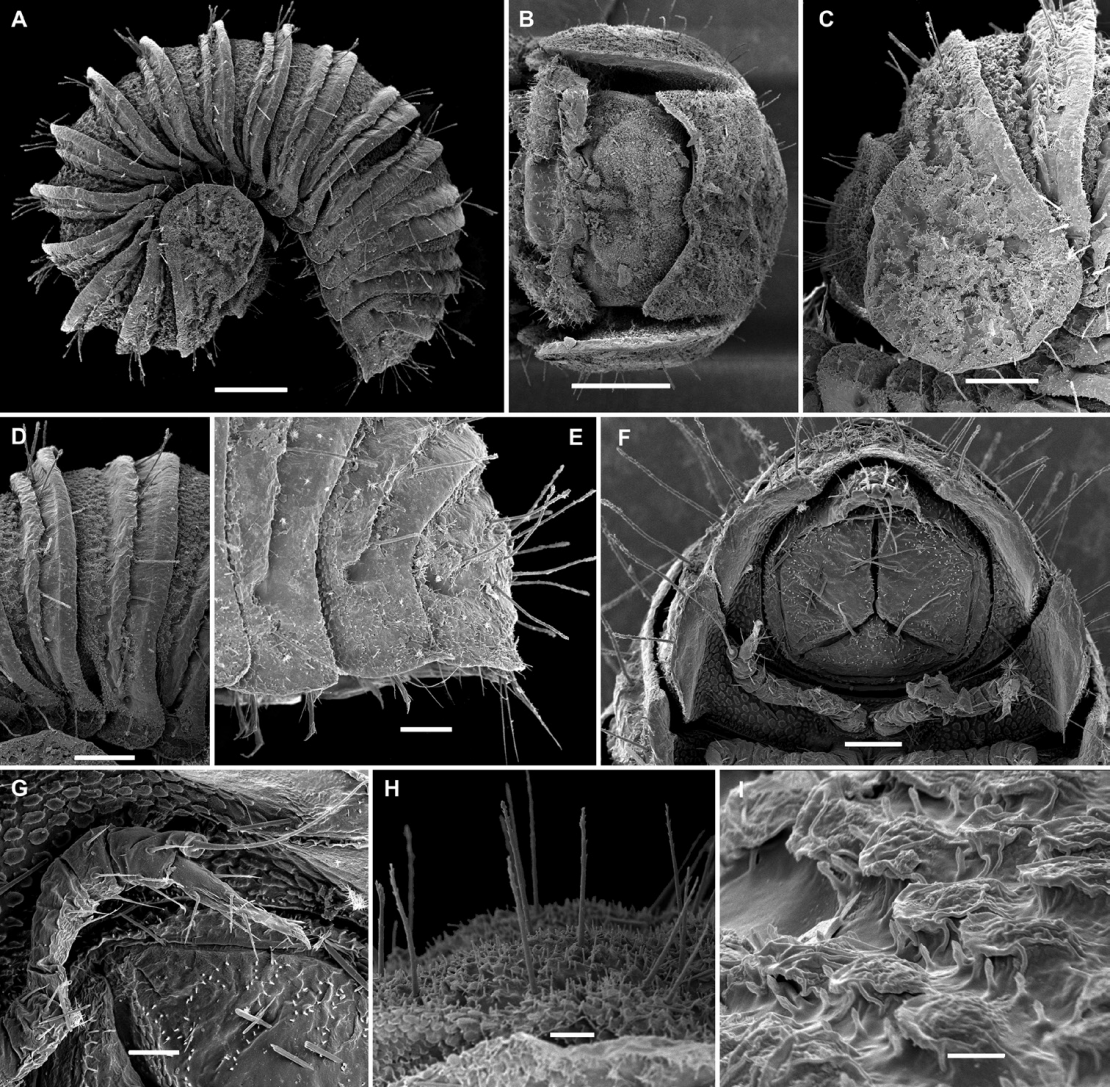
*Ammodesmus congoensis* sp. n., a 19-segmented ♀ paratype. **A** habitus, lateral view **B** head and collum, dorsal view **C, D, E** anterior, middle and caudal parts of body, respectively, lateral view **F** posterior part of body, caudal view **G** last left leg, lateral view **H** tergal setae, dorsal view **I** tegument texture, dorsal view. Scale bars: **A, B** = 200 µm; **C, D** = 100 µm; **E, F** = 50 µm; **G, H** = 20 µm; **I** = 5 µm.

#### Remarks.

The total absence of ozopores in *Ammodesmus congoensis* sp. n. is well documented by Fig. 1A which shows an adult ♂ with 18 segments clean enough to be certain. In both other *Ammodesmus* species, in addition to the pits near the base of the paraterga, which are also observed in the new species, ozopores are clearly visible closer to the ventral ends of paraterga 5, 7, 9, 10, 12, 13, 15–17(18). A similar pore formula is observed in *Elassystremma* species: 5, 7, 9, 12, 13, 15–17(18). As variation in ozopore distribution in Ammodesmidae is a proven fact, we are reluctant to create a separate, new genus for *Ammodesmus congoensis* sp. n. only because it totally lacks ozopores. The most important character that brings the new species together with *Ammodesmus granum* and *Ammodesmus nimba* is the strangely modified last leg-pair.

Variation in adults of both sexes showing either 18 or 19 segments is another piece of evidence bringing *Ammodesmus congoensis* sp. n. especially close to *Ammodesmus granum*. The latter has hitherto remained the only species of Polydesmida where the number of segments varies regardless of sex. As in the ♂ the size of the body likewise correlates positively with gonopod size (Fig. 1I), an additional molt of the 18-segmented adult turning it into the 19-segmented one is obviously implied.

### Key to *Ammodesmus* species

**Table:** 

1	Metaterga smooth, either densely pilose and/or sparsely setose (Figs 1A–F, 3A, D). Gonopods simple, telopodites biramous (Figs 1I, J, 2A–C). Central Africa	***Ammodesmus congoensis* sp. n.**
–	A transverse row of large, flat, low bosses or high tubercles located in caudal 1/3 of metaterga, the latter neither setose nor pilose. Gonopods different. Western Africa	**2**
2	No sexual dimorphism in tergal sculpture. Gonopods complex, with extremely large coxae concealing telopodites inside a deep gonocoel	***Ammodesmus nimba***
–	Drastic sexual dimorphism in tergal sculpture: ♂ with, ♀ without, high tubercles across metaterga. Gonopods simple, telopodites uniramous, mostly represented by solenomeres	***Ammodesmus granum***

## Conclusion

**Figure 4.**
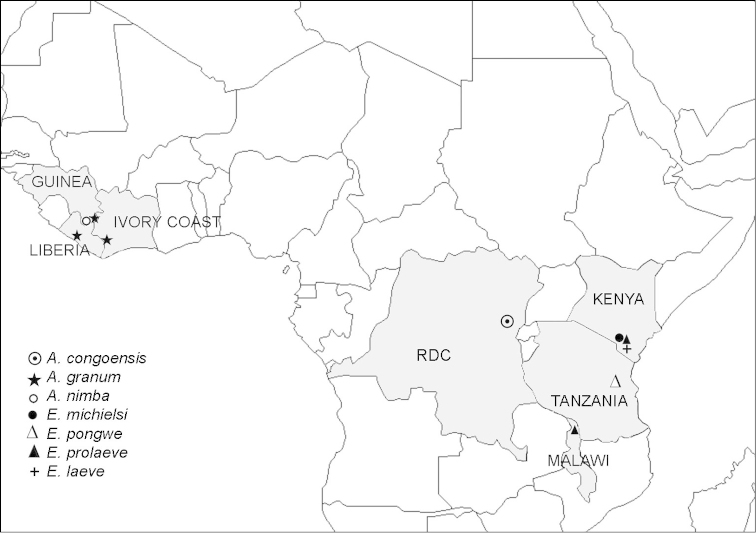
Updated distribution of the family Ammodesmidae.

Fig. 4 shows an updated distribution of the known species of Ammodesmidae. *Ammodesmus* is more widespread, occurring both in western and central Africa. Only *Ammodesmus granum* is relatively widespread, whereas the other two congeners seem to be highly local in distribution. In contrast, *Elassystremma* seems to be confined to eastern Africa. All four *Elassystremma* species (*Elassystremma pongwe* Hoffman & Howell, 1981, *Elassystremma michielsi* VandenSpiegel & Golovatch, 2004, *Elassystremma laeve* VandenSpiegel & Golovatch, 2004 and *Elassystremma prolaeve* VandenSpiegel & Golovatch, 2004) are slightly larger than *Ammodesmus* (up to 5 mm long), and their gonopods are invariably complex, sunken inside a deep gonocoel (VandenSpiegel and Golovatch 2004). Likewise, only one species, *Elassystremma prolaeve*, is widespread, occurring not only in Kenya and Malawi, but probably also in between in Tanzania.

## Supplementary Material

XML Treatment for Ammodesmus congoensis
